# Toxicity Mechanisms of Cigarette Smoke on Mouse Fetus Mitochondria

**Published:** 2015

**Authors:** Parvaneh Naserzadeh, Mir-Jamal Hosseini, Baharak Mohamadzadeh Asl, Jalal Pourahmad

**Affiliations:** a *School of Pharmacy, Shahid Beheshti University of Medical Sciences, Tehran, Iran.*; b *Zanjan Applied Pharmacology Research Center, Zanjan University of Medical Sciences, Zanjan, Iran.*

**Keywords:** Cigarette smoke extract (CSE), Embryo toxicity, Mouse fetus, Isolated mitochondria

## Abstract

Maternal smoking has been recognized as a common cause of low birth weight, preterm birth and the decrease of gestational age period. Unfortunately, there is an increasing interest within public especially woman in Iran in the tobacco products consumption. On the other hand, the deleterious effect of maternal smoking on human fetus in pregnancy period especially in the first trimester encouraged us to investigate toxicity mechanisms of cigarette smoke on mouse fetus mitochondria. For this purpose different concentrations (1, 10 and 100%) of standardized cigarette smoke extract (CSE) were administrated on mitochondria isolated from fetus of NMRI mice on the 15 day of gestation. Our results showed a significant increase in ROS (Reactive oxygen species) formation, lipid peroxidation, mitochondrial membrane potential collapse, mitochondrial swelling and finally a decrease in ATP concentration in the CSE-treated isolated fetus mitochondria. Our results suggest that CSE-induced embryo toxicity is the result of disruptive effect on mitochondrial respiratory chain that leads to ROS formation, lipid peroxidation, mitochondrial MMP (mitochondrial membrane potential) decline and decrease of ATP level which starts apoptosis signaling.

## Introduction

Maternal smoking has been recognized as a common cause of low birth weight, preterm birth and decrease of the gestational age period (1, 2). Although there is evidence to suggest a dose-response relationship between the number of cigarettes smoked during pregnancy and the decline in fetus birth weight such as twice the rate of low birth weight infants in African-American mothers in the United States compared with white mothers in different socioeconomic stratum (3, 4). Several studies suggested that race, ethnicity, modifiable behavioral factors may have a more strongly negative effect on fetal growth in maternal tobacco abuse (5). Besides, maternal smoking is related to variety of respiratory and vascular diseases, and cancer (6). Importance of cigarette smoke is due to 158 known toxic chemicals such as formaldehyde, acrolein, polycyclic aromatic hydrocarbons, nitrosamines, arsenic, cadmium and chromium which can be linked with various diseases and cancers (7, 8).

Previous studies on different tissues of rat such as liver, skin, heart and brain showed involvement of oxidative stress in toxicity mechanisms of cigarette smoke which is responsible for most of the damages at the origin of cell and isolated mitochondria (6). Barnoya and Glantz suggested that cigarette smoke caused increasing risk of coronary heart disease via platelet activation, enhanced oxidative stress, reduced antioxidant defense, induction of endothelial dysfunction and inflammation similar with our results (9). Other studies in *in-vivo* animal model suggested increasing of ROS formation, lipid peroxidation and glutathione-S-transferase (GST) activity which confirms involvements of oxidative stress in cigarette smoking (10). Unfortunately, There is increasing interest of public specially woman in Iran in the tobacco products consumption. On the other hand, the effect of maternal smoking on the fetus in pregnancy period especially in the first trimester caused to investigate toxicity mechanisms of cigarette smoke in mouse fetus. According to the latest information in the database there are only a few articles which have studied the toxicity effects of cigarette smoke on fetuses on mammals. Therefore, it planned to study the toxic mechanisms of cigarette smoke in isolated mitochondria obtained from fetuses’ rat precisely by measuring different mitochondrial toxic parameters in mouse fetus.

## Experimental

*Materials*

All chemicals and reagents were purchased from Sigma-Aldrich (Taufkrichen, Germany) in the best commercial grade. Aqueous cigarette smoking extract was known as 100% CSE and used at lower concentrations (1, 10, and 100%) by diluting 100% CSE in dionized water. 

*Cigarette smoke extract preparation*

Cigarette smoke extract (CSE) was standardized by bubbling the smoke from one 1R5F research grade cigarette (1.67 mg tar, 0.16 mg nicotine, and 2.08 mg total particulate matter per cigarette; The University of Kentucky, Lexington, KY) into 10 mL of RPMI 1640 with 10% FBS over 3 min using a cigarette smoking apparatus. The CSE was pH corrected (7.4), then the filter was sterilized and the absorbance value was read at 320 nm using a Tecan GENios plate reader (Basel, Switzerland), and only CSE preparations with an absorbance value of 0.42 ± 0.03 were used. The resulting CSE was known as 100% CSE and used at lower concentrations (1, 10, and 100%) by diluting 100% CSE in RPMI 1640 with 10% FBS. Vehicle control medium was made by bubbling air through RPMI 1640 with 10% FBS for 3 min followed by filter sterilizing. 

*Animals *

The animals used in this research, were mice of NMRI race, purchased from Institute Pasteur (Tehran, Iran). All mice were fed with a normal standard chow diet and tap water ad libitum. All experiments were conducted according to the ethical standards and protocols approved by the Committee of Animal Experimentation of Shahid Beheshti University of Medical Sciences, Tehran, Iran. To investigate the effect of cigarette smoke extract (CSE) on rat fetus mitochondria, Mitochondria were isolated from differential centrifugation and CSE % concentrations (1, 10 and 100) were then be applied. The animals were anesthetized at 15 day of gestation. Following laparatomy, the uterus was exteriorized and fetuses randomly were examined carefully for determination of toxicity mechanisms cigarette smoke on fetuses affected by maternal smoking. Then, total of fetus were rapidly rinsed using isotonic saline buffer. These samples were used for the isolation of mitochondria as described below. 

*Preparation of mitochondria*

Mitochondria were prepared from differential centrifugation and homogenized with a glass hand held homogenizer with previous method (11, 12) Protein concentration was determined by the Coomassie blue protein-binding method using BSA (bovine serum albumin), as the standard sample (12, 13). 

*In-vitro evaluation of mitochondrial parameters*

The mitochondrial ROS production was assayed by F-2500 fluorescence spectrophotometer (HITACHI) using DCFH-DA (2',7'-dichlorofluorescin diacetate) in the period of 60 min (14, 15, 16). The activity of mitochondrial complex II (succinate dehydrogenase) was determined by measuring the reduction of MTT (3-(4,5-dimethylthiazol-2-yl)-2,5-diphenyltetrazolium bromide) (12, 14). The content of the malondialdehyde (MDA), the lipid peroxidation marker was assessed by measuring the absorbance of the supernatant at 532 nm with an ELISA reader as described in previous study (15, 17). Reduced glutathione (GSH) level was determined in mitochondrial extracts using DTNB (dithiobis-2-nitrobenzoic acid) reagent using by spectrophotometer. GSH content was expressed as µg/mg protein (16, 18). Mitochondrial membrane potential was determined by mitochondrial uptake of rhodamine 123 with fluorescence spectrophotometer at the excitation and emission wavelength of 490 nm and 535 nm, respectively (12, 15, and 18). Mitochondrial swelling was assayed using a previously reported method by monitoring the absorbance at 540 nm (12, 19, and 20). Finally, the ATP level and ATP/ADP ratio were measured by luciferase enzyme (12, 21).

*Statistical analysis*

Results are presented as means ± SD. All statistical analyses were performed using the SPSS software, version 17. Assays were performed in triplicate and the mean was used for the statistical analysis. Statistical significance was determined using the one-way ANOVA test, followed by the post-hoc Tukey test. Statistical significance was set at P < 0.05.

## Results

**Figure 1:**
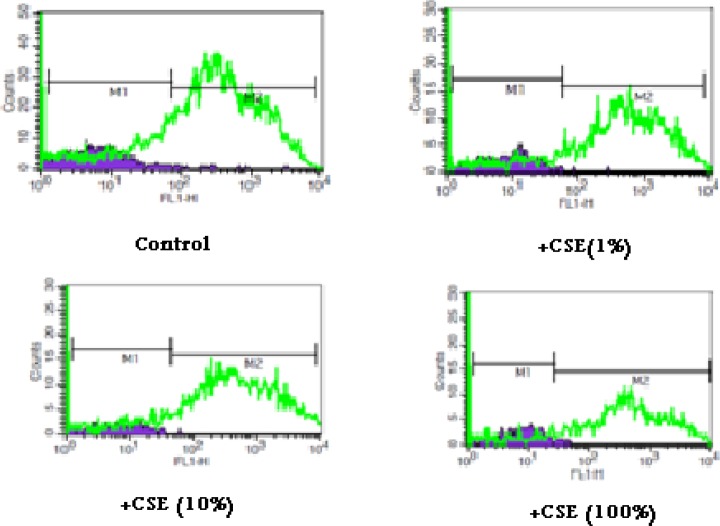
ROS formation in aqueous cigarette smoking extract (CSE) treated fetus mitochondria**. **ROS formation was evaluated after addition of CSE% concentration (0,1,10 and 100) after 15 min incubation .ROS formation was determined by flowcytometry using DCF-DA as described in materials and methods. FL1: the fluorescence intensity of DCF.

**Figure 2:**
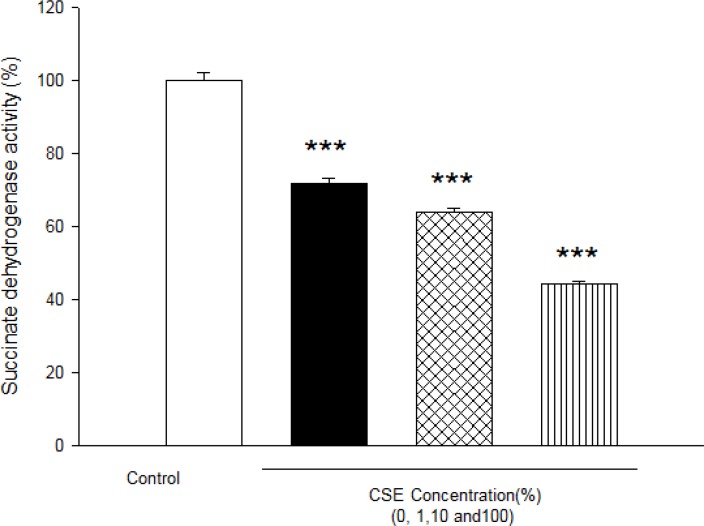
Effect of aqueous cigarette cigarette smoking extract (CSE) on Succinate dehydrogenase activity.Succinate dehydrogenase activity was measured using MTT dye as described in Materials and methods. Isolated mitochondria (0.5 mg/mL) were incubated for 1h with various concentrations of CSE% concentration (0, 1, 10 and 100). Values represented as mean±SD (n=3). * ***P<0.001 compared with control mitochondria from fetus mitochondria.

**Figure 3:**
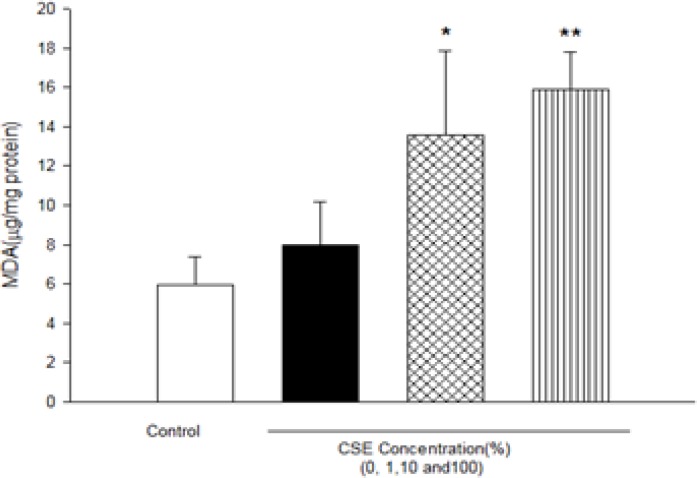
Effect of aqueous cigarette smoking extract on lipid peroxidation on isolated fetus mitochondria. Isolated mitochondria (0.5 mg/mL) were incubated for 1h with various concentrations of aqueous CSE%concentration (0,1 ,10 and 100) .Values represented as mean±SD (n=3). *P<0.05 ; **P<0.01 compared with control mitochondria.

As shown in Figure 1, CSE concentration (1, 10 and 100%) induced significant ROS formation rise on fetus mitochondria which were measured with two methods: fluorescence spectrophotometer using DCFH-DA and flowcytometry assay. As demonstrated in Table 1 and Figure 1, there is a significant concentration dependent shift of DCF peak to the right ward and increasing of ROS production in a concentration–dependent manner. Succinate dehydrogenase (complex II) activity was also assessed using the MTT test after 1 h incubation of mitochondria obtained from fetus with different CSE concentrations (1, 10 and 100%). As shown in Figure 2, all concentrations of CSE could significant decrease in the mitochondrial metabolic conversion of MTT to formazon (p<0.05). As shown in Figure 3, only 1% concentration of CSE could not a significant increase of MDA in isolated fetus mitochondria compared to control mitochondria. But, CSE concentrations (10% and 100%) caused significant MDA production on the isolated fetus mitochondria compared to control groups.

**Table 1:** Aqueous cigarette smoking extract (CSE) induced ROS formation on isolated fetus mitochondria.

**Groups**	**ROS**				
	**5 5 min**	**15 min**	**30 min**	**45 min**	**60 min**
**Fetus**					
**Control**	0±1	6±2	9±1	15±2	19±2
**+CSE (1%)**	4±2	42±6*	55±14	60±5*	64±8
**+CSE (10%)**	7±2*	108±12***	113±29**	132±17***	143±22***
**+ CSE (100%)**	8±4*	173±18***	183±31***	196±29***	220±33***

ROS formation was determined by fluorescence spectrophotometer using DCFH-DA as described in materials and methods and demonstrated as fluorescence intensity of DCF. Values represented as mean±SD (n=3).

* *P*<0.05;

** *P*<0.01;

*** *P*<0.001 compared with control mitochondria at the same time interval

**Figure 4:**
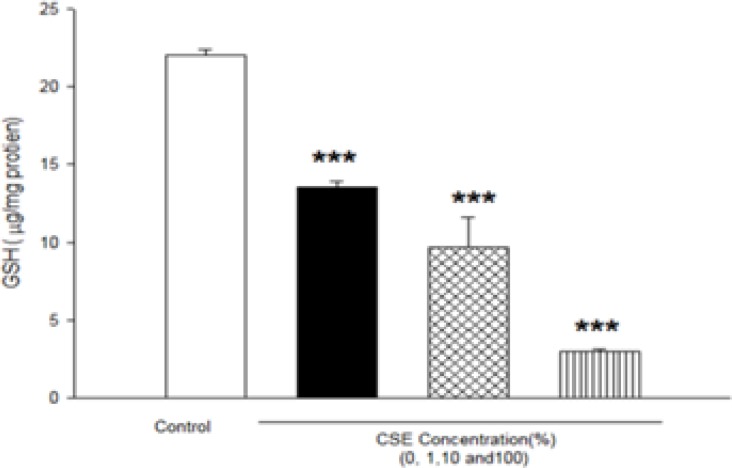
Effect of aqueous cigarette smoking extract on mitochondrial GSH content in isolated fetus mitochondria. Isolated mitochondria (0.5 mg/mL) were incubated for 1h with various concentrations of aqueous CSE%concentration (0,1 ,10 and 100) .Values represented as mean±SD (n=3). ***P<0.001 compared with control mitochondria.

As shown in Figure 4, GSH level after incubation of fetus mitochondria with CSE concentrations (1, 10 and 100%) significantly decreased compared to control mitochondria (P<0.05). 

**Figure 5:**
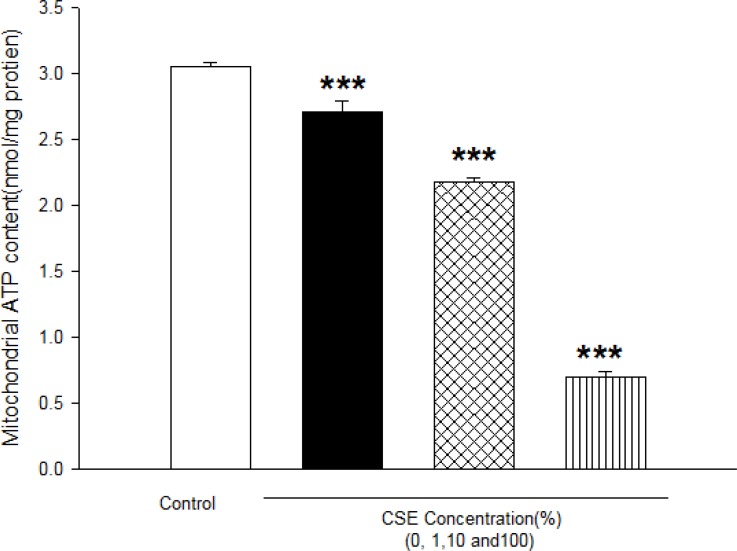
Effect of aqueous cigarette smoking extract on mitochondrial ATP level. Isolated mitochondria (0.5 mg/mL) were incubated with CSE% concentrations (0,1,10 and 100) and ATP level were determined using *Luciferin /Luciferase *Enzyme System as described in Materials and methods. Values represented as mean±SD (n=3). ***P<0.001 compared with control mitochondria.

A s shown in Table 2, CSE concentrations (1, 10 and 100%) significantly decreased the MMP in all the mitochondrial test groups in a concentration and time dependent model (p<0.05)(Table 2). As shown in Table 3, addition of CSE concentrations (1, 10 and 100%) to isolated mitochondrial leads to mitochondrial swelling without undetermined manner as indicator of mitochondrial membrane permeability transition (MPT) (Table 3). As shown in Figure 5, CSE concentrations (1, 10 and 100%) significantly decreased mitochondrial ATP levels in isolated fetus mitochondria in a concentration dependent manner as indicator of mitochondrial dysfunction. 

**Table 2:** Effect of aqueous cigarette smoking extract on mitochondrial membrane potential MMP collapse (∆Ψ%) on isolated fetus mitochondria.

**Groups**	**∆Ψ%**				
	**5 5 min**	**15 min**	**30 min**	**45 min**	**60 min**
**Fetus**					
**Control**	0±1	0±1	18±3	38±2	49±2
**+CSE (1%)**	12±2*	12±2*	115±25***	107±12***	119±12***
**+CSE (10%)**	18±5**	18±5**	240±23***	255±21***	276±19***
**+ CSE (100%)**	23±6***	23±6***	300±27***	315±34***	349±16***

Mitochondrial membrane potential collapse (**∆**Ψ%) was measured by Rhodamine 123 as described in Materials and Methods. The effect of aqueous CSE concentration% (0, 1, 10 and 100) on the mitochondrial membrane potential decrease in fetus mitochondria was evaluated. The values are expressed as means ± SD (n=3). Values represented as mean±SD (n=3).

* P<0.05;

** *P*<0.01;

*** *P*<0.001 compared with control mitochondria.

**Table 3:** Effect of aqueous cigarette smoking extract (CSE) on the mitochondrial swelling

**Groups**	**Mitochondrial Swelling percent (%)**				
	**5 5 min**	**15 min**	**30 min**	**45 min**	**60 min**
**Fetus**					
**Control**	0±1	19±1	39±5	72±3	84±3
**+CSE (1%)**	4±1*	49±3**	52±4*	77±3	89±4
**+CSE (10%)**	9±2***	48±8**	56±2**	80±3	90±2
**+ CSE (100%)**	29±1***	48±9**	66±4***	88±4**	92±5

Mitochondrial swelling was measured by determination of absorbance at 540 nm as described in Materials and methods. Values represented as mean±SD (n=3).

* P<0.05;

** *P*<0.01;

*** *P*<0.001 compared with control mitochondria.

## Discussion

Mitochondria are important organelles with major role in cellular function, ATP production, regulating energy expenditure, apoptosis signaling, and production of reactive oxygen species (20). Recently, analysis of mitochondrial function is used in diagnosis of many diseases including the cancer, diabetes, cardiovascular disease, oxidative stress, and the age-related neurodegenerative diseases (20). Therefore, it decided to evaluated toxicity mechanism of cigarette smoke in fetus in pregnant mothers. Epidemiological reports suggested that cigarette smoke contains many known potent carcinogens, that increases the oxidative burden of the cell, which when persisted may lead to many pathological conditions (22, 23). 

It has been reported that exposure to environmental tobacco smoke for pregnant women also results in the reduction of the fetal bi parietal diameter during weeks 20–24 of gestation , low birth weight and an increased risk of spontaneous abortion (22, 23). Our previous studies in cigarette smoke suggested that mitochondria play a key role in the apoptosis of cells by CSE via decline in the antioxidant activity and increasing of ROS formation accompanied with the loss of mitochondrial membrane potential and decreasing of ATP level (7, 11). These results are warranting data for public health which smoking cessation or avoidance of exposure with cigarette smoke in crowded places. Our key finding in previous study suggested internal tissue (brain and heart) have high sensitivity compared with external tissue (skin) (6). In present study, rapid increase in ROS formation following addition of CSE on isolated fetus mitochondria confirmed the probable involvement of mitochondrial ROS in CSE induced toxicity mechanisms (Figure 1 and Table 2). Furthermore, significant reduction in complex II activity as indicator of decreased mitochondrial oxidative capacity (24), increasing of LPO and glutathione reduction in fetus mitochondria represented oxidative stress as one of the pathophysiological mechanisms of cigarette smoke toxicity. Our data in fetus mitochondria also corroborated with the previous findings, which showed that free radicals and ROS generated by CSE may play a key role in the instigation of membrane LPO (6, 7). It suggested that oxidation of mitochondrial lipid membranes could result in disruption of mitochondrial electron transfer chain and consequently collapse of mitochondrial membrane potential and mitochondrial swelling which both events lead to increased ROS formation and disturbance in mitochondrial electron transfer. Besides, GSH (reduced glutathione) oxidation in MPT pores could damage to mitochondrial membrane integrity and opening of MPT pores, leading to cell death signaling (15, 19). Therefore, decreasing of GSH level in present study caused impairing in protection of cellular antioxidant system and direct action on mitochondrial ATPase activity and cytochrome C oxidase rather than through oxygen free radical injury (12, 15, 24 and 25). Finally, addition of CSE to isolated fetus mitochondria will inevitably decrease the proton motive force in mitochondria and then inhibition of ATP synthesis and inhibition of cytochrome oxidase (25, 26). Our data also showed similar results with previous studies which related in impairment of ETC in ability of ATP synthesis. In addition, depletion in ATP levels promotes a switch from apoptotic to necrotic cell death, however, ATP depletion more than 80% leads to necrosis due to down regulation of an ATP synthase subunit (24, 25, and 26). Mitochondrial depolarization or MPT (mitochondrial permeability transition) pores opening would be expected to cause ATP depletion due to conversion of mitochondrial ATP syntase to ATPase, followed by substantial mitochondrial swelling.

The previous studies about precise mechanism of cigarette extract toxicity suggested that oxidative stress plays a key role in vital tissues. Present investigation also confirmed impairment in electron transfer chain leading to increased ROS production, failure of oxidative phosphorylation, rapid increase in mitochondrial membrane lipid peroxidation, mitochondrial swelling and finally decline of cellular ATP which can then promote cell death signaling. Besides, it seems this is unique study to address CSE -induced oxidative stress and its consequences on isolated fetus mitochondria which can help to better understanding of CSE toxicity mechanism.
